# Supplementary material for: Roles of metal ions in the selective inhibition of oncogenic variants of isocitrate dehydrogenase 1
Source: Commun Biol. 2021 Nov 1;4:1243. doi: 10.1038/s42003-021-02743-5 (PMC8560763; doi:10.1038/s42003-021-02743-5)
Supplement: Supplementary file 2 — Supplementary Information [file 42003_2021_2743_MOESM2_ESM.pdf]

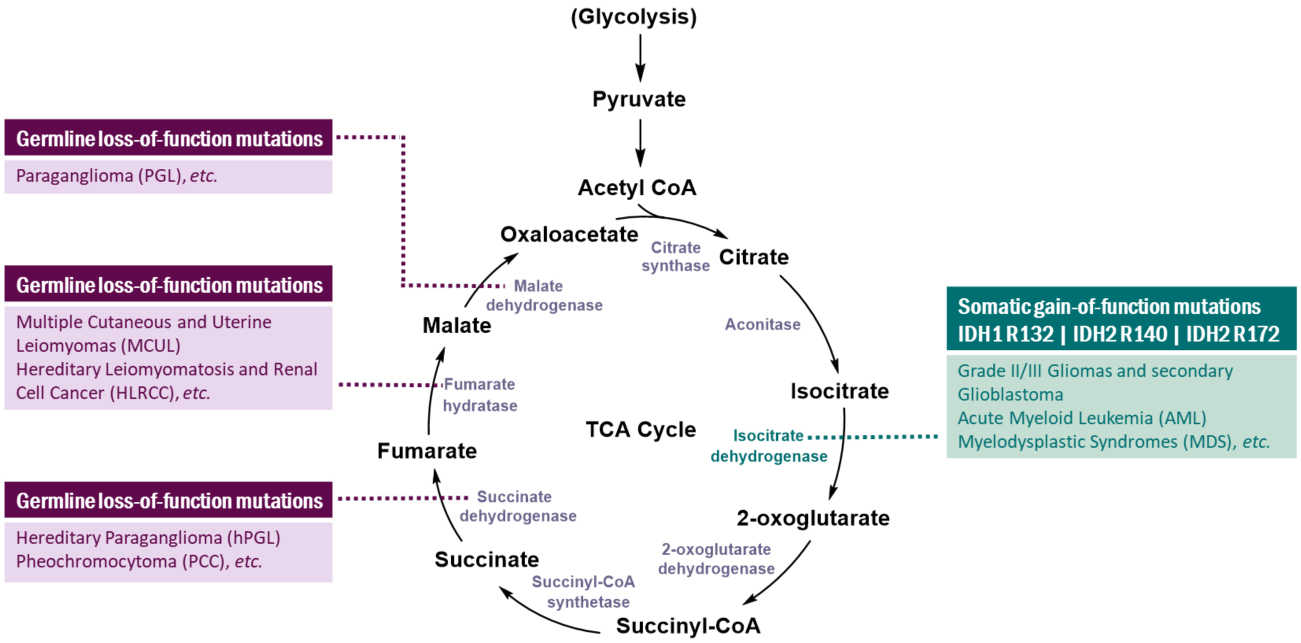

Supplementary Figure S1. **Summary of mutations to genes ending for tricarboxylic acid cycle enzymes linked to cancer**. Malate dehydrogenase, fumarate hydratase and succinate dehydrogenase genes undergo germline loss-of-function mutations associated with some rare cancers[1–3]. Isocitrate dehydrogenases genes undergo somatic gain-of-function mutations as exemplified by IDH1 R132, IDH2 R140 and IDH2 R172 variants, some of which are drug targets for glioma, glioblastoma and acute myeloid leukemia[4].

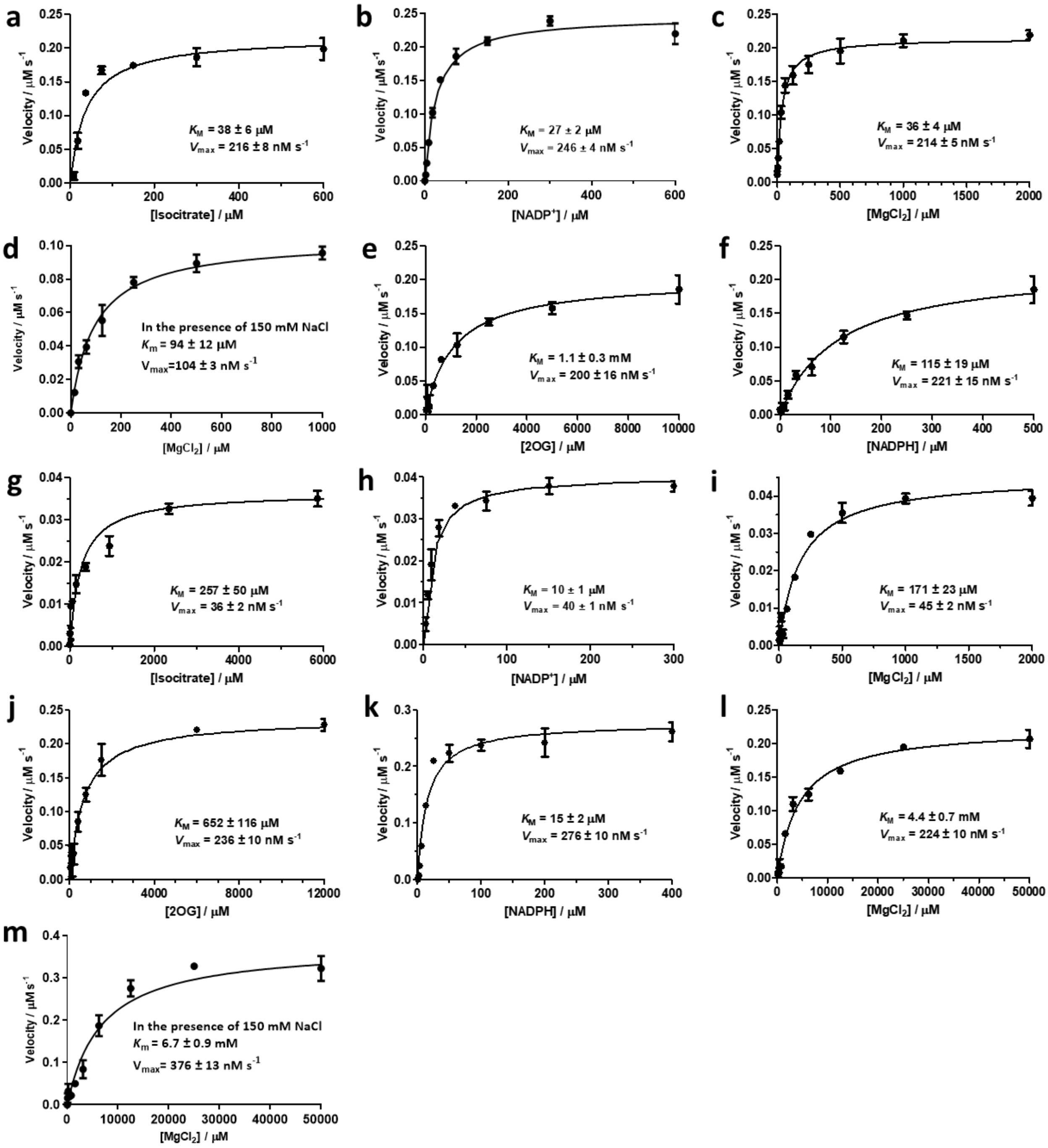

Supplementary Figure S2. Dependence of wt IDH1 (a—f) or R132H IDH1 (g—m) reaction rates (monitored by NADPH appearance/disappearance) on concentrations of cosubstrates, substrates and Mg^{2+} ions. **(a)** DL-isocitrate, **(b)** $NADP^+$, **(c)** $MgCl_2$, **(d)** $MgCl_2$ in the presence of 150 mM NaCl, for oxidation of isocitrate to 2OG by wt IDH1. **(e)** 2OG, **(f)** NADPH for reduction of 2OG to isocitrate by wt IDH1. Kinetic parameters for $MgCl_2$ during reduction of 2OG to isocitrate by wt IDH1 could not be determined as reaction occurs without added $MgCl_2$. **(g)** DL-isocitrate, **(h)** $NADP^+$, **(i)** $MgCl_2$ for oxidation of isocitrate to 2OG by R132H IDH1. **(j)** 2OG, **(k)** NADPH, **(l)** $MgCl_2$, **(m)** $MgCl_2$ in the presence of 150 mM NaCl, for reduction of 2OG to 2HG by R132H IDH1. Buffer: 100 mM Tris-HCl, 10 mM $MgCl_2$ (except for kinetic parameters

determination of $MgCl_2$), 0.005% (v/v) Tween 20, 0.1 mg/mL BSA and 0.2 mM DTT, pH 8.0. See Methods for assay conditions. Error bars represent mean ± SEM, n= 3 technical replicates. The calculated K_M and V_{max} values are reported as mean ± SEM from the data fit.

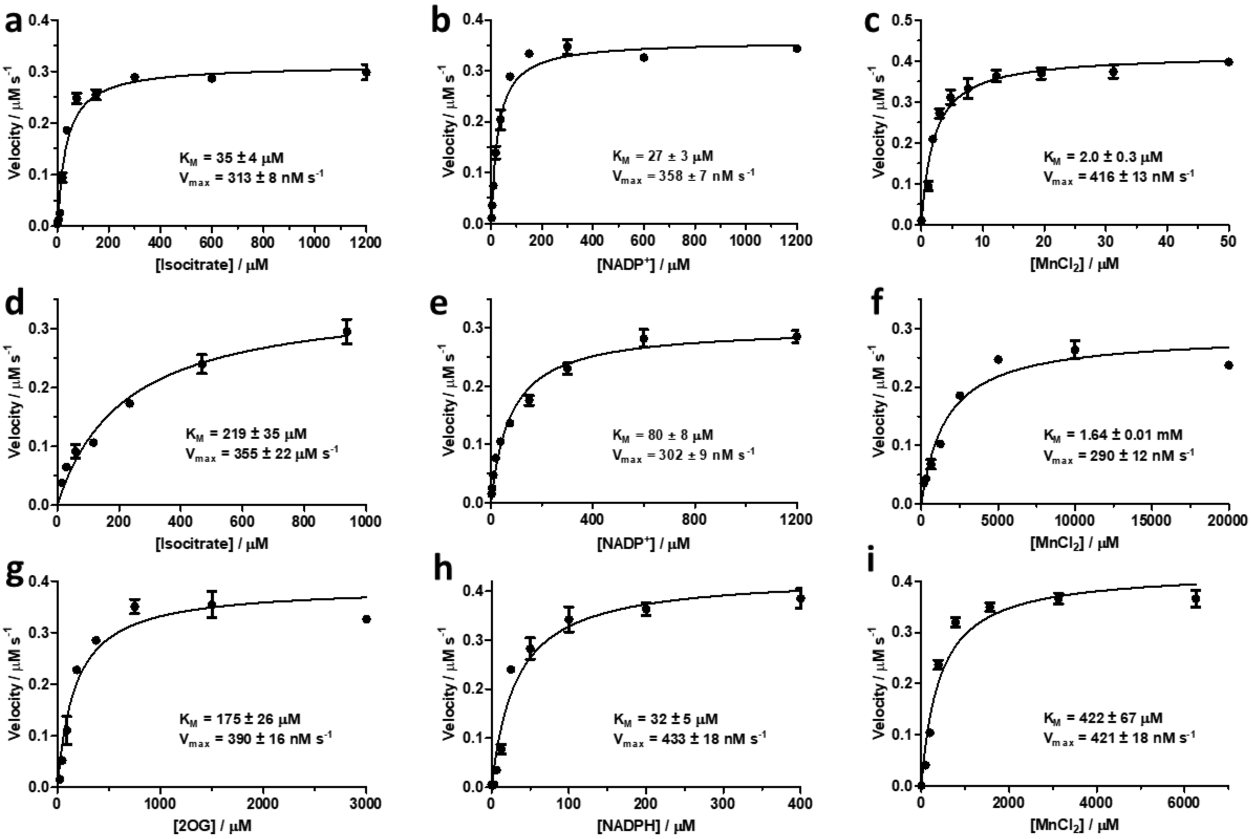

Supplementary Figure S3. Dependence of wt IDH1 (a—c) or R132H IDH1 (d—i) rates (monitored by NADPH appearance/disappearance) on concentrations of cosubstrates, substrates, and Mn^{2+} ions. (a) DL-isocitrate, **(b)** $NADP^+$, **(c)** $MnCl_2$ for the oxidation of isocitrate to 2OG by wt IDH1. Kinetic parameters for reduction of 2OG to isocitrate by wt IDH1 were not determined as $MnCl_2$ forms insoluble carbonate salt with $NaHCO_3$ in the reaction mixture. **(d)** DL-isocitrate, **(e)** $NADP^+$, **(f)** $MnCl_2$ for the oxidation of isocitrate to 2OG by R132H IDH1. **(g)** 2OG, **(h)** NADPH, **(i)** $MnCl_2$ for the reduction of 2OG to 2HG by R132H IDH1. Buffer: 100 mM Tris-HCl, 10 mM $MnCl_2$ (except for kinetic parameters determination of $MnCl_2$), 0.005% (v/v) Tween 20, 0.1 mg/mL BSA and 0.2 mM DTT, pH 8.0. See Methods for assay conditions. Error bars represent mean ± SEM, n= 3 technical replicates. The calculated K_M and V_{max} values are reported as mean ± SEM from the data fit.

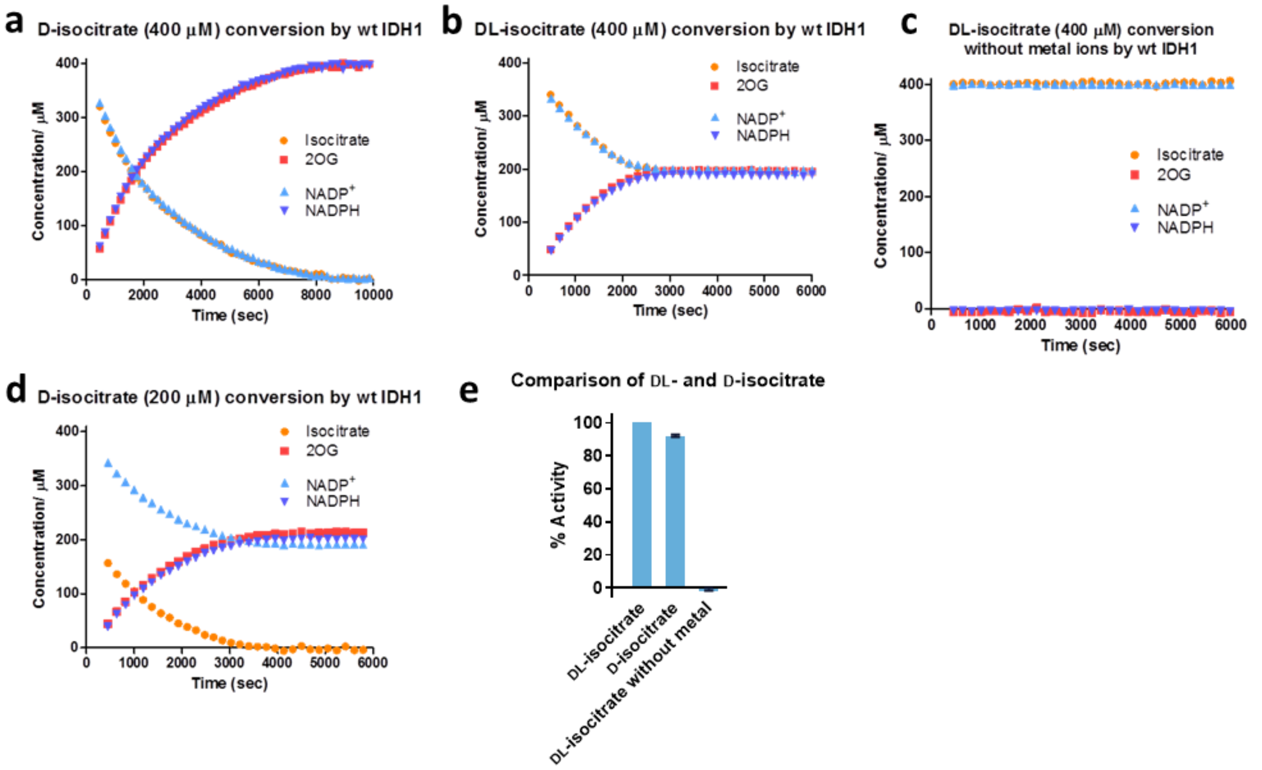

Supplementary Figure S4. **L-Isocitrate is neither a substrate nor an inhibitor of wt IDH1.** Conversion of DL-/ D-isocitrate and $NADP^+$ to 2OG and NADPH by wt IDH1, as monitored by ^1H NMR (700 MHz). Concentrations of D-isocitrate and DL-isocitrate were normalised to equal signal intensity prior to reaction monitoring, as DL-isocitrate has an undefined water content in the commercial product. See Methods for details. **(a)** D-isocitrate was fully converted. **(b)** DL-isocitrate was ~50% converted, because only D-isocitrate reacts. **(c)** DL-isocitrate was not converted in the absence of metal ions. **(e)** Comparing **(b)** and **(d)** which contain equal concentration of D-isocitrate (200 mM), the rates of wt IDH1 catalysed conversion of isocitrate to 2OG are similar, *i.e.* the conversion of D-isocitrate is not inhibited by L-isocitrate.

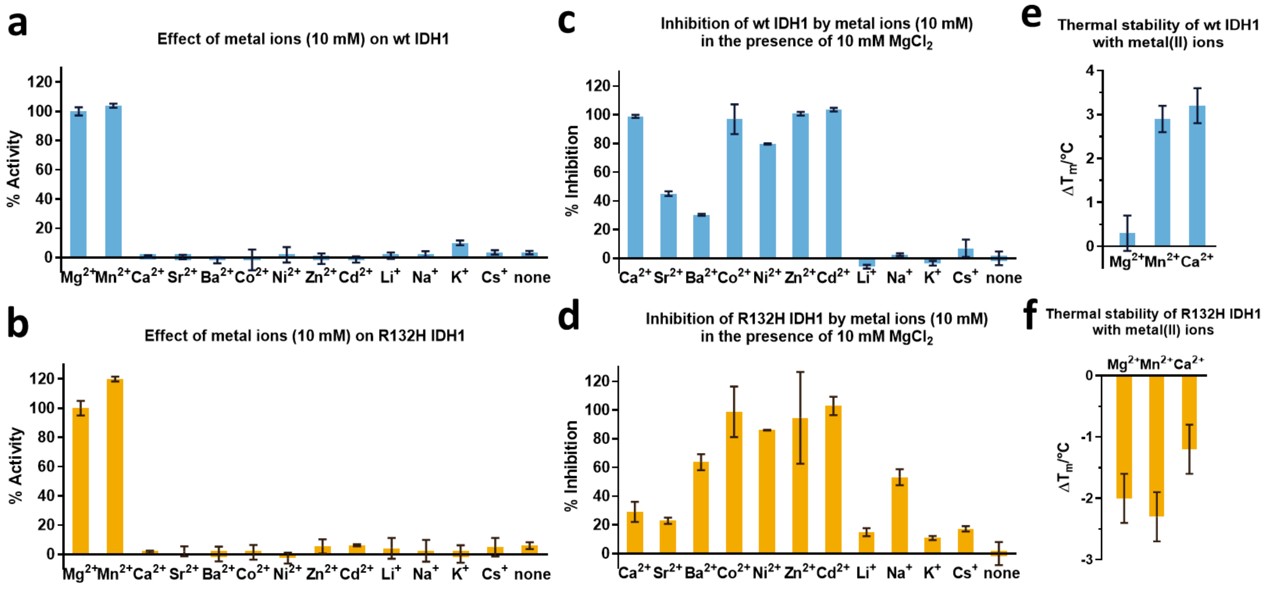

Supplementary Figure S5. Activities and thermal stabilities of wt IDH1 and R132H IDH1 with different metal ions. Effect of metal ions (10 mM) on catalysis by **(a)** wt IDH1 and **(b)** R132H IDH1; inhibition of **(c)** wt IDH1 and **(d)** R132H IDH1 by metal ions (10 mM) in the presence of 10 mM $MgCl_2$, as measured by absorbance-based assays. See Methods for assay conditions. Data are mean ± SD, n= 3 technical replicates. Chloride salts were used in all cases. Activities were normalised to that observed with $MgCl_2$. A no-enzyme control was subtracted from the raw data for $CoCl_2$ due to the intrinsic absorbance of Co^{2+} in buffer. Mn^{2+} and less commonly, Ni^{2+} and Co^{2+}, can sometimes be used as surrogates for Mg^{2+}. Mn^{2+} can replace Mg^{2+} in many enzymes using MgATP as the substrate, resulting in similar / sometimes higher enzymatic activity[5]. Ca^{2+} is generally a poor substitute for Mg^{2+} due to its significantly larger ionic radius. These results show that wt and R132H IDH1 are active with Mg^{2+} or Mn^{2+}. In general, inhibition was observed with other divalent metal ions and no activity with monovalent metal ions. However, it is inconclusive that divalent transition metals (Co^{2+}, Ni^{2+}) and post-transition metals (Zn^{2+}, Cd^{2+}) act through direct inhibition. Cd^{2+}, for instance, was observed to cause IDH1 precipitation in NMR experiments. Thermal shift results of divalent metal ions (5 mM) addition for **(e)** wt IDH1 or **(f)** R132H IDH1 in 50 mM Tris-HCl, pH 7.5, as measured by DSF. Data are mean ± SD, n= 3 technical replicates. In general, divalent metal ions appear to stabilise wt IDH1 but destabilise R132H IDH1.

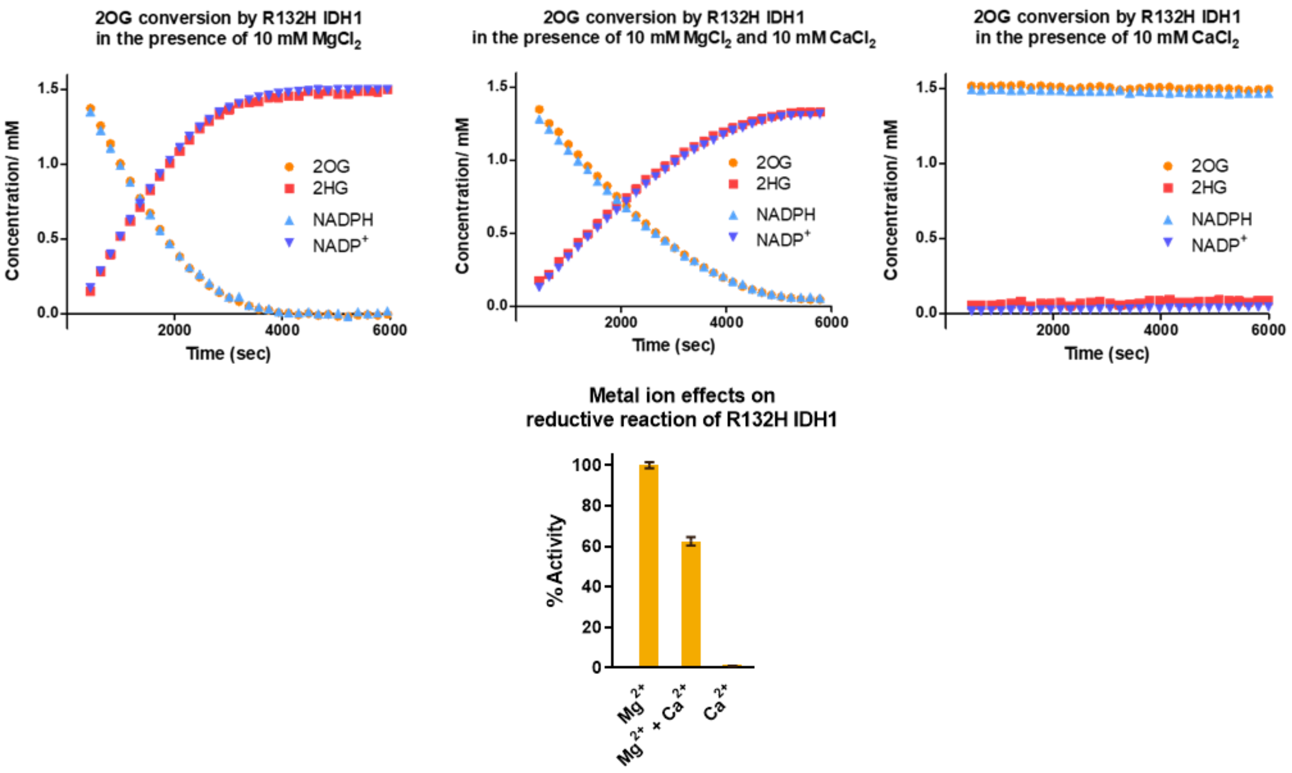

Supplementary Figure S6. **Ca^{2+} is not catalytically active and inhibits R132H IDH1.** Inhibition (equimolar as the active metal ion Mg^{2+}) and activity of 10 mM $CaCl_2$ on R132H IDH1 catalysed conversion of 2OG to 2HG, as monitored by ^{1}H NMR (700 MHz). See Methods for details.

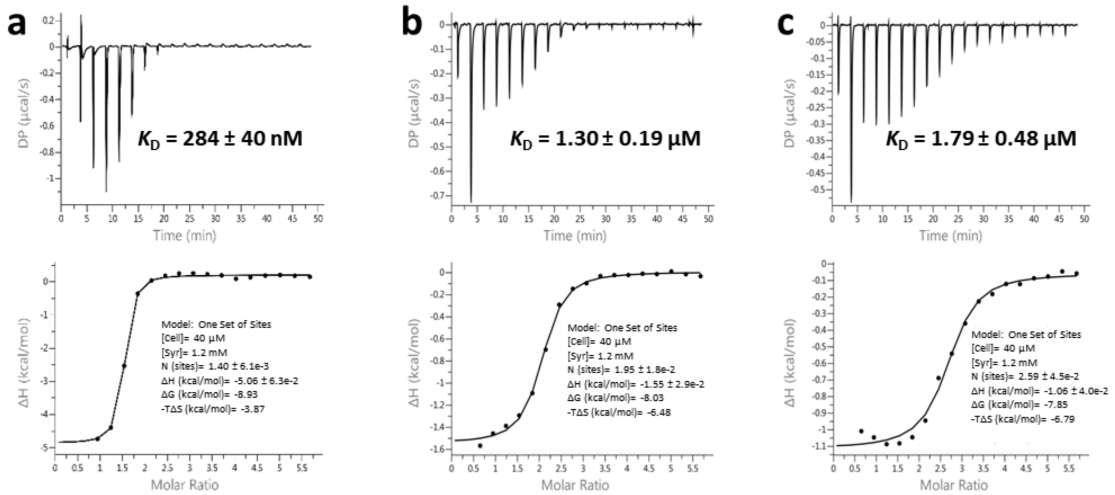

Supplementary Figure S7. **ITC analysis of DL-isocitrate binding to wt IDH1 in the presence of (a) 5 mM $MgCl_2$ and 150 mM NaCl, (b) 5 mM $MnCl_2$ and (c) 5 mM $CaCl_2$, in 50 mM Tris-HCl, pH 7.5**. See Methods for details. The results show that isocitrate-Mg^{2+} binds to wt IDH1 with nanomolar affinity in the presence of 150 mM NaCl; isocitrate-Mn^{2+} and isocitrate-Ca^{2+} bind to wt IDH1 with low micromolar affinity.

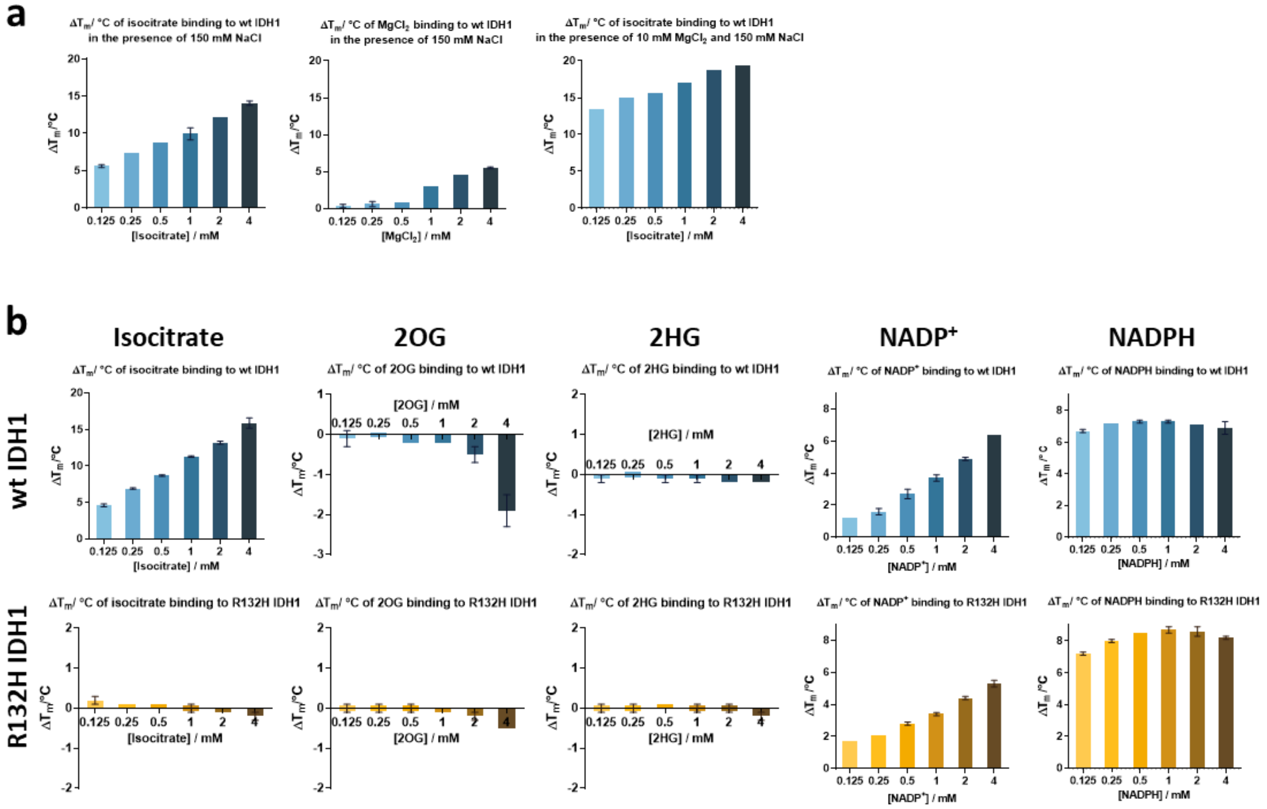

Supplementary Figure S8. **Dose-dependent (0.125—4 mM) thermal stabilisation of wt or R132H IDH1 by cosubstrates. (a)** DSF results for DL-isocitrate, $MgCl_2$ and DL-isocitrate in the presence of 10 mM $MgCl_2$, with wt IDH1 in 50 mM Tris-HCl, 150 mM NaCl, pH 7.5. Thermal stabilisation of wt IDH1 by isocitrate is enhanced by $MgCl_2$ in the presence of 150 mM NaCl. $MgCl_2$ itself induces weak thermal shifts. **(b)** DSF results for DL-isocitrate, 2OG, 2HG, $NADP^+$ and NADPH, with wt or R132H IDH1 in 50 mM Tris-HCl, pH 7.5. The results show isocitrate, $NADP^+$, and NADPH stabilise wt IDH1, whilst only $NADP^+$ and NADPH are observed to stabilise R132H IDH1. Data are mean ± SD, n= 3 technical replicates. See Methods for details.

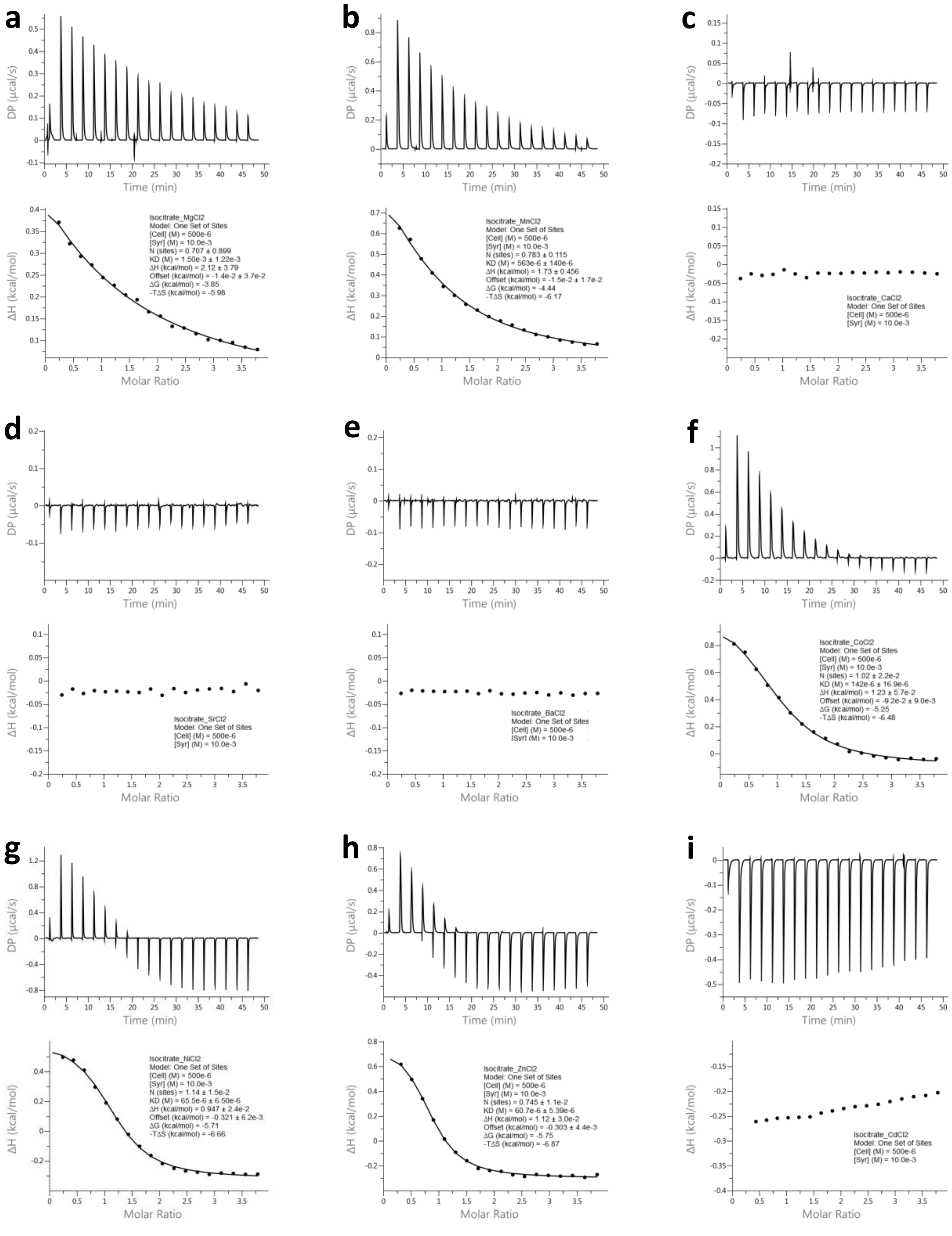

Supplementary Figure S9. **ITC analysis of metal ion (10 mM) binding to DL-isocitrate (500 µM) in 50 mM Tris-HCl, pH 7.5.** See Methods for details. Metal ions: **(a)** $MgCl_2$, **(b)** $MnCl_2$, **(c)** $CaCl_2$, **(d)** $SrCl_2$, **(e)** $BaCl_2$, **(f)** $CoCl_2$, **(g)** $NiCl_2$, **(h)** $ZnCl_2$, **(i)** $CdCl_2$. Rank order of binding affinity for isocitrate: $ZnCl_2$ (strongest)> $NiCl_2$ > $CoCl_2$ > $MnCl_2$ > $MgCl_2$; no detected binding of $CaCl_2$, $SrCl_2$, $BaCl_2$, and $CdCl_2$. The K_D values appear to correlate with the ionic radii of metal ions and

consequently the bond distance between the metal ions and oxygen atoms of isocitrate; metal ions with ionic radii ≥95 pm and M–O distance ≥2.30 Å (*i.e.* Cd^{2+}, Ca^{2+}, Sr^{2+} and Ba^{2+}) showed no binding to isocitrate (**Figure 2g**).

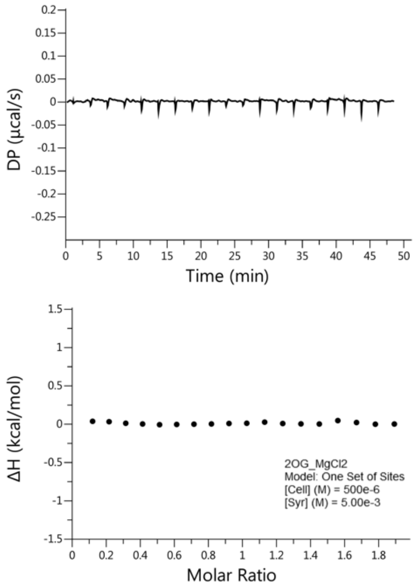

Supplementary Figure S10. **ITC analysis of $MgCl_2$ (5 mM) binding to 2OG (500 µM) in 50 mM Tris-HCl, pH 7.5.** See Methods for details. No binding is detected between Mg^{2+} and 2OG.

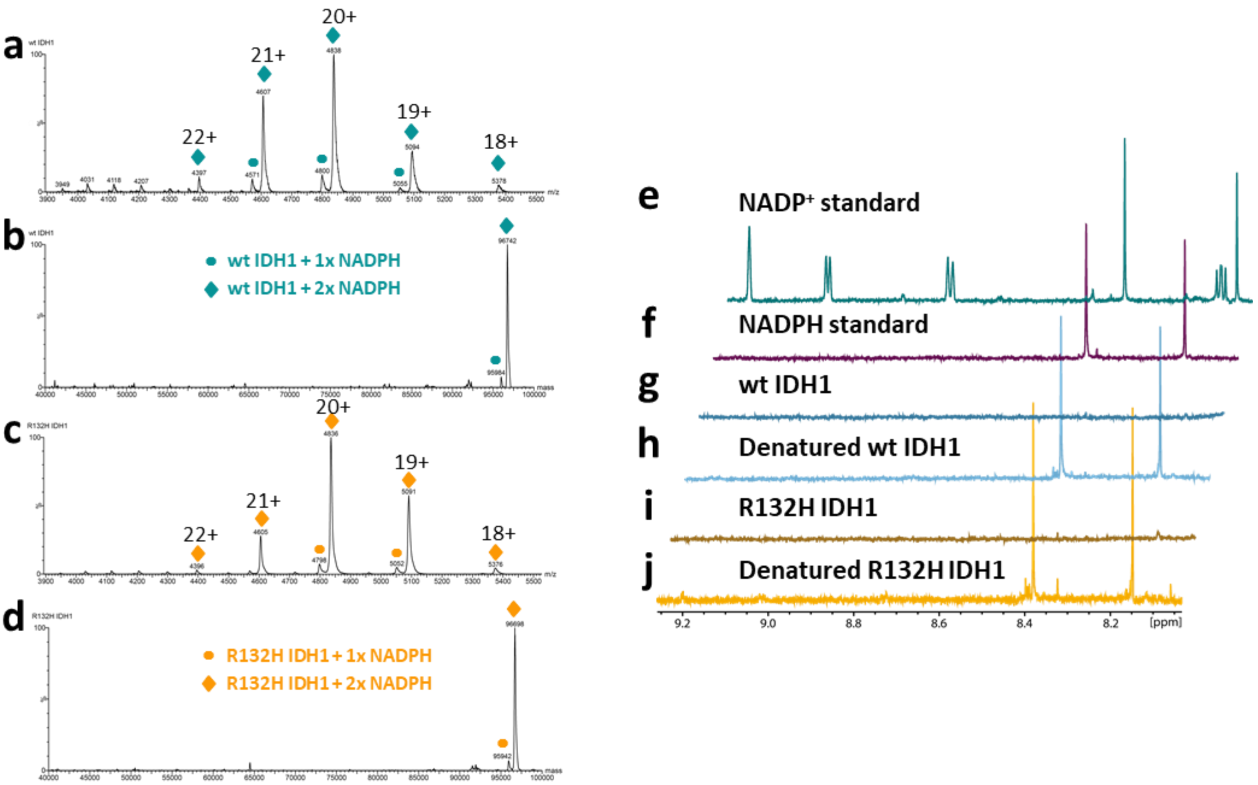

Supplementary Figure S11. **wt and R132H IDH1 copurify with NADPH.** Raw (charge state distribution of 18^+ to 22^+) and deconvoluted non-denaturing mass spectra of 50 µM **(a**, **b)** wt IDH1 and **(c**, **d)** R132H IDH1 showing a dimer complexed with two copurifying NADPH molecules. Cone voltage: 200 V. Theoretical mass of monomeric wt or R132H IDH1= 47.7 kDa. Observed mass 96.7 kDa = theoretical dimer protein mass without *N*-terminal Met + 2× NADPH. See Methods for details. CPMG-edited ^{1}H NMR spectra to identify the copurifying cofactor in wt and R132H IDH1. **(e)** $NADP^+$ (50 µM) in D_2O. **(f)** NADPH (50 µM) in D_2O. **(g)** wt IDH1 (50 µM) in D_2O. Neither $NADP^+$ nor NADPH are observed as cofactor is tightly bound to the protein. **(h)** wt IDH1 denatured by heating **(g)** at 90 °C for 30 s. The precipitate was removed; the supernatant contains released NADPH. **(i)** R132H IDH1 (50 µM) in D_2O. Neither $NADP^+$ nor NADPH can be observed as cofactor is tightly bound to the protein. **(j)** Denatured R132H IDH1 produced by heating at 90 °C for 30 s. The precipitate was removed; the supernatant contains released NADPH.

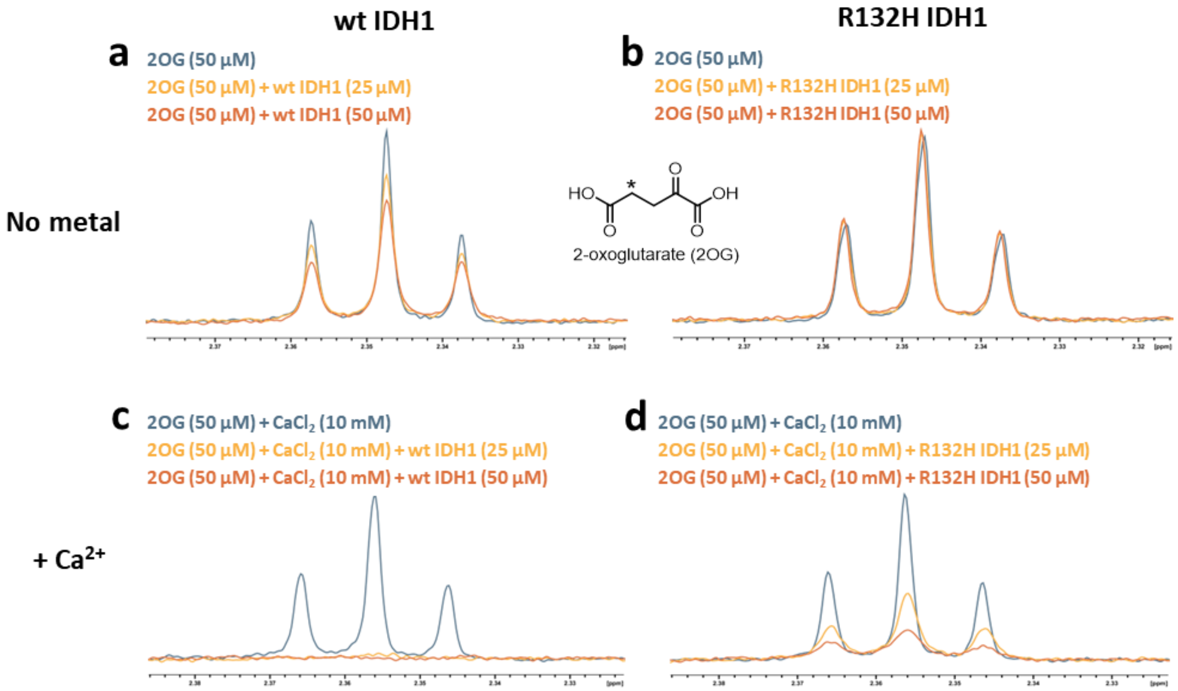

Supplementary Figure S12. **2OG binding to R132H IDH1 is enhanced by Ca^{2+} ions.** CPMG-edited ^{1}H NMR analyses of 2OG binding to **(a)** wt IDH1 (0.5 and 1 molar equivalents compared to 2OG) and **(b)** R132H IDH1 (0.5 and 1 molar equivalents compared to 2OG) in the absence of divalent metal ions, and 2OG binding to **(c)** wt IDH1 and **(d)** R132H IDH1 in the presence of 10 mM $CaCl_2$. Buffer: 50 mM Tris-D_{11}-HCl, pH 7.5 in 90% H_2O/ 10% D_2O (v/v). Blue: substrate or substrate-metal complex; Yellow-orange: substrate or substrate-metal complex with 0.5 molar equivalent protein. Orange: substrate or substrate-metal complex with equimolar protein. See Methods for details. $CaCl_2$ enhances 2OG binding to both wt and R132H IDH1. In particular, no binding of 2OG to R132H IDH1 was detected without divalent metal ions, but partial binding was achieved after Ca^{2+} addition. 2OG binds to wt IDH1 more strongly than to R132H IDH1, with or without divalent metal ions.

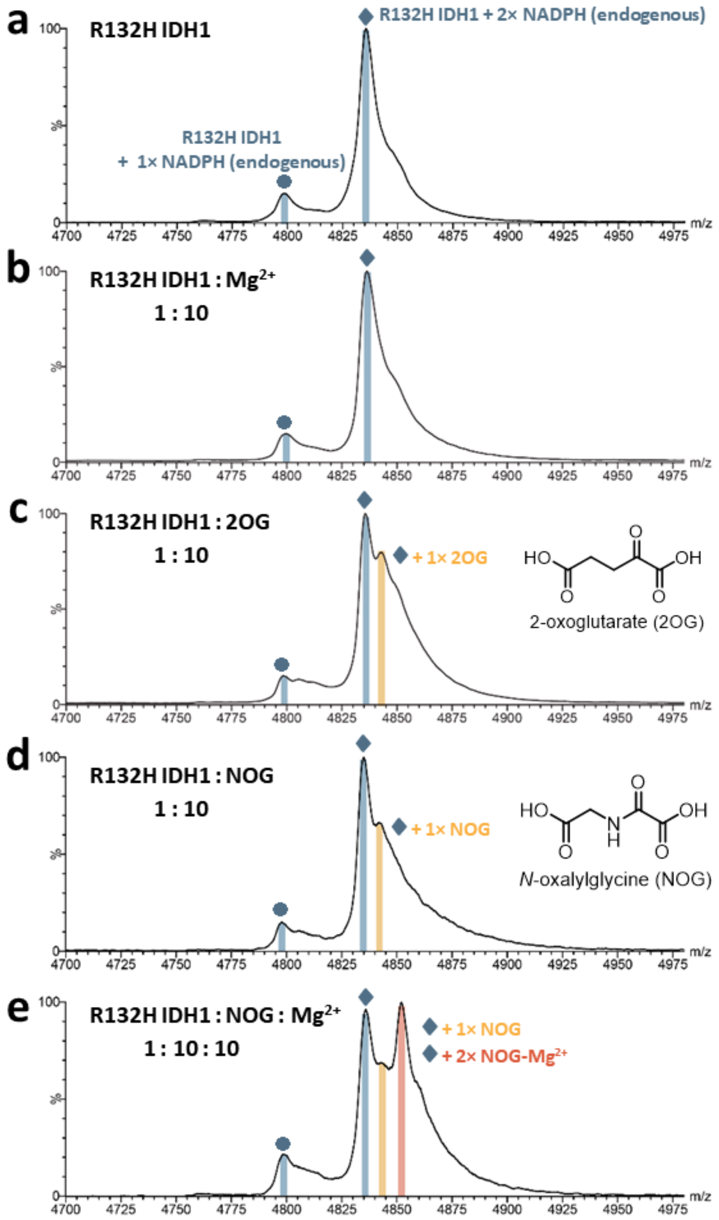

Supplementary Figure S13. **Binding of NOG (a 2OG analogue) to R132H IDH1 is enhanced by Mg^{2+} ions.** Non-denaturing MS analysis of EDTA-treated R132H IDH1 binding to 2OG/NOG, in the absence or presence of Mg^{2+}. Data for the IDH1 dimer m/z= 20^{+} charge state are shown. Cone voltage: 100 V unless otherwise stated. Buffer: 200 mM ammonium acetate, pH 7.5. See Methods for details. **(a)** R132H IDH1 (50 µM) exists as a dimer with 2 copurifying NADPH molecules. **(b)** R132H IDH1+$MgCl_2$ (50:500 µM). On addition of a 10 fold molar excess of $MgCl_2$, the mass increases slightly, possibly due to non-specific binding. **(c)** R132H IDH1+2OG (50:500 µM) showing weak binding of one 2OG molecule to IDH1 dimer. **(d)** R132H IDH1+NOG (50:500 µM) indicating weak binding of one NOG molecule to the IDH1 dimer, similar to **(c)**. **(e)** R132H IDH1+NOG+$MgCl_2$ (50:500:500 µM). Addition of Mg^{2+} leads to the IDH1 dimer complexed with 2 NOG molecules.

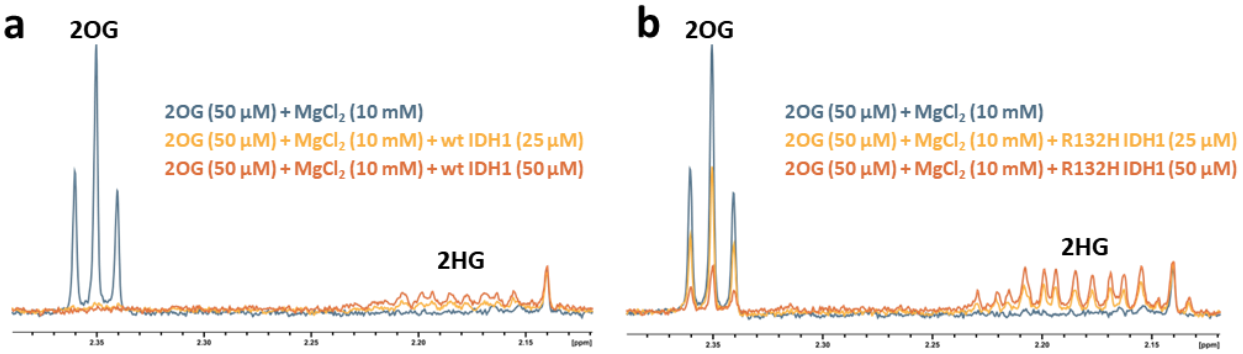

Supplementary Figure S14. **CPMG-edited ^{1}H NMR binding analyses of 2OG binding to (a) wt IDH1 and (b) R132H IDH1 in the presence of Mg^{2+}.** Buffer: 50 mM Tris-D_{11}-HCl, pH 7.5 in 90% H_2O/ 10% D_2O (v/v). Blue: substrate or substrate-metal complex; Yellow-orange: substrate or substrate-metal complex with 0.5 molar equivalent protein. Orange: substrate or substrate-metal complex with equimolar protein. See Methods for details. The loss of the 2OG signal is (partly) due to its conversion to 2HG by wt and R132H IDH1.

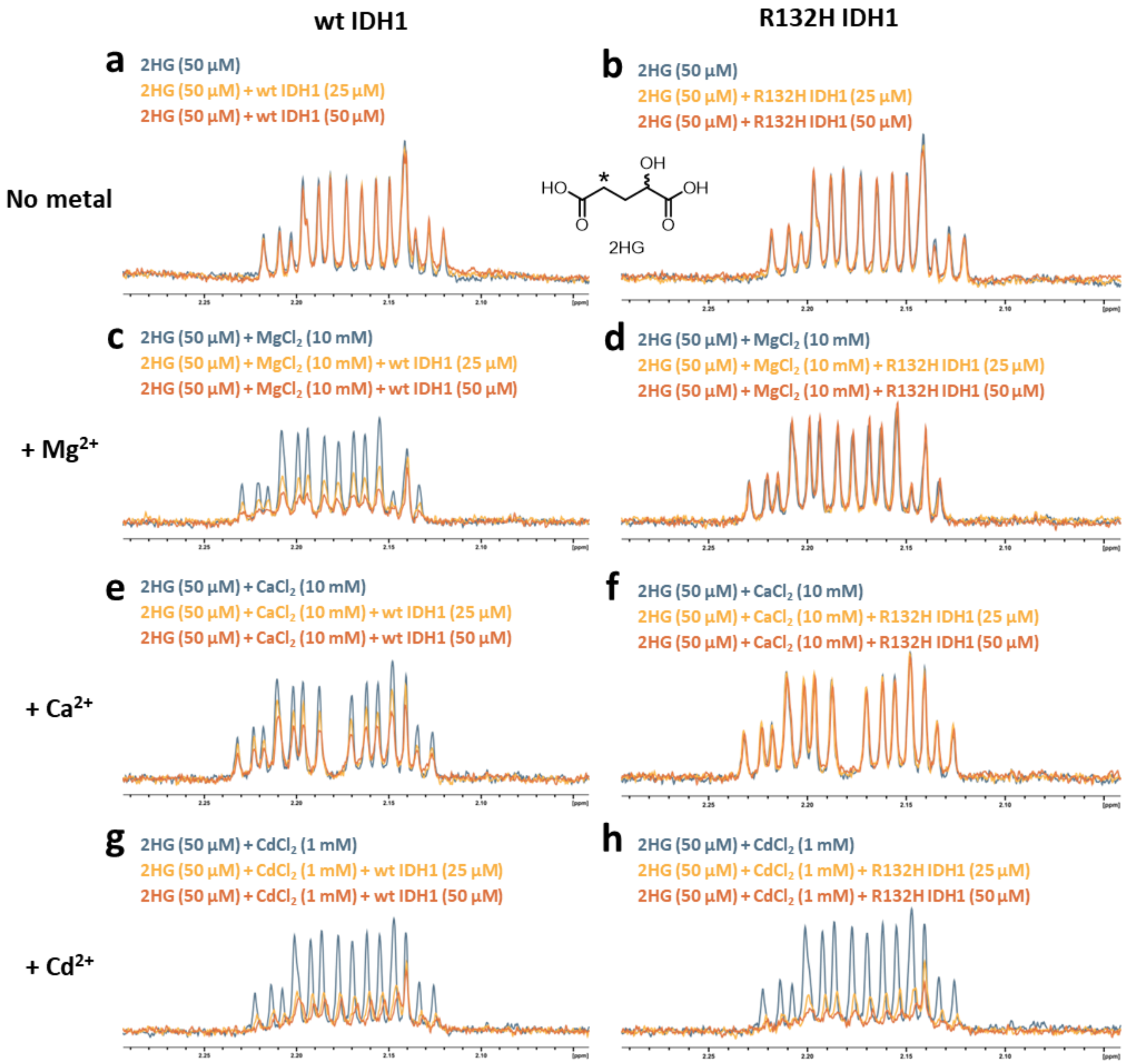

Supplementary Figure S15. **Metal ions ($MgCl_2$, $CaCl_2$, $CdCl_2$) promote 2HG binding to wt IDH1 and R132H IDH1.** CPMG-edited ^{1}H NMR binding analyses of 2HG binding to **(a, c, e, g)** wt IDH1 and **(b, d, f, h)** R132H IDH1 in the absence of metal ions, in the presence of Mg^{2+} (10 mM), Ca^{2+} (10 mM) and Cd^{2+} (1 mM) respectively. Note, $CdCl_2$, in particular, promotes 2HG binding to R132H IDH1. Buffer: 50 mM Tris-D_{11}-HCl, pH 7.5 in 90% H_2O/ 10% D_2O (v/v). Blue: substrate or substrate-metal complex; Yellow-orange: substrate or substrate-metal complex with 0.5 molar equivalent protein. Orange: substrate or substrate-metal complex with equimolar protein. See Methods for details. Note that Cd^{2+} was added at a lower concentration (1 mM) than for Mg^{2+} and Ca^{2+} (10 mM), as it was observed to cause significant precipitation at higher concentrations.

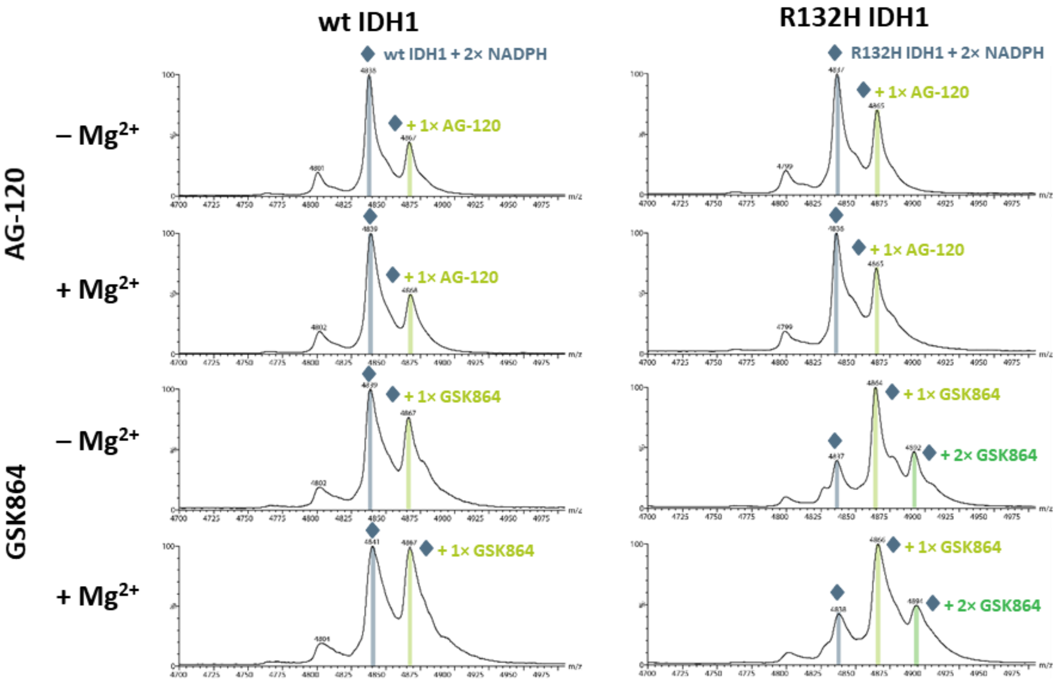

Supplementary Figure S16. **Binding of AG-120 or GSK864 to wt IDH1/R132H IDH1 are similar with and without $MgCl_2$.** Non-denaturing MS analysis of binding of AG-120 or GSK864 to EDTA-treated wt IDH1 or R132H IDH1, ± $MgCl_2$. Data for the IDH1 dimer m/z= 20^+ charge state are shown. Cone voltage: 100 V. Buffer: 200 mM ammonium acetate, pH 7.5. See Experimental Methods for details. For clarity, only 2× NADPH bound protein peaks are annotated. Samples for wt IDH1 contain 50 µM wt IDH1, 12.5 µM AG-120 or 25 µM GSK864, ± 200 µM $MgCl_2$. Samples for R132H IDH1 contain 50 µM R132H IDH1, 12.5 µM AG-120 or 50 µM GSK864, ± 500 µM $MgCl_2$.

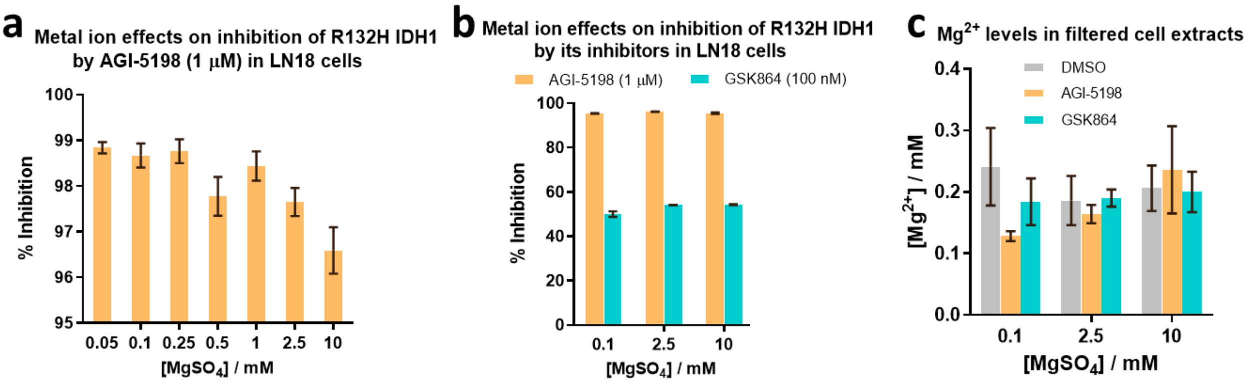

Supplementary Figure S17. Studies on R132H IDH1 inhibition in LN18 cells in the presence of different Mg^{2+} concentrations. (a) R132H IDH1 catalysis in LN18 cells is inhibited by AGI-5198 (1 μM) at all tested $MgSO_4$ concentrations, but the extent of inhibition does not decrease significantly with increasing $MgSO_4$, as determined by intracellular 2HG levels relative to DMSO control. Data are mean ± SD, n= 3 biological replicates. **(b)** R132H IDH1 catalysis in LN18 cells is inhibited by AGI-5198 (1 μM) and GSK864 (100 nM) at all tested $MgSO_4$ concentrations; no clear correlation between the inhibition extent and $MgSO_4$ concentration is observed. Data are mean ± SD, n= 3 biological replicates. **(c)** Intracellular Mg^{2+} levels in cell lysates of **(b)** are similar across all samples despite different added $MgSO_4$ concentrations in the media, as measured by cation exchange chromatography with conductivity detection (CEC-CD). Mg^{2+} concentrations reported represent those in the filtered and dsDNA normalised cell extracts, harvested using 200 μL 80% MeOH from LN18 cells cultured in 6 cm dishes. Data are mean ± SD, n= 3 biological replicates. See Experimental Methods for details.

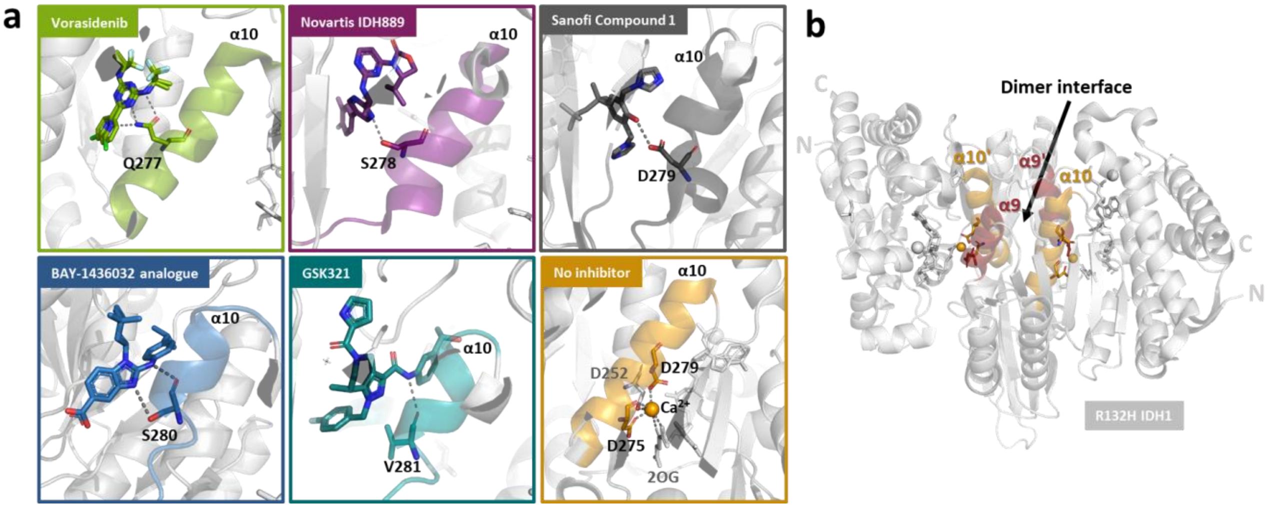

Supplementary Figure S18. **IDH inhibitors form hydrogen bonds with α10 of R132H IDH1, one face of which is required for metal ion coordination. (a)** Crystal structure views of R132H IDH1-inhibitor complexes. Inhibitors and α10 (residues N271-G286) are coloured for clarity, with the inhibitor-interacting residues shown in sticks and hydrogen bonds shown in grey dotted lines: Vorasidenib with Q277 (green, PDB 6ADG)[6], Novartis IDH889 with S278 (purple, PDB 5TQH)[7], Sanofi 1 with D279 (dark grey, PDB 4UMX)[8], BAY-1436032 analogue with S280 (navy, PDB 5LGE)[9], and GSK321 with backbone of V281 (teal, PDB 5DE1)[10]. In the absence of inhibitors (orange, PDB 3INM)[11], D275 and D279 of α10 (orange sticks), together with D252 from α9' of the other monomer and substrate 2OG (light grey sticks), coordinate to inhibitory Ca^{2+} (orange sphere, substituting for Mg^{2+}). **(b)** The dimer interface of the R132H IDH1 homodimer[11] is formed predominantly by α9 (red, residues I251—K260) and α10 (orange, residues N271—G286) from each monomer subunit.

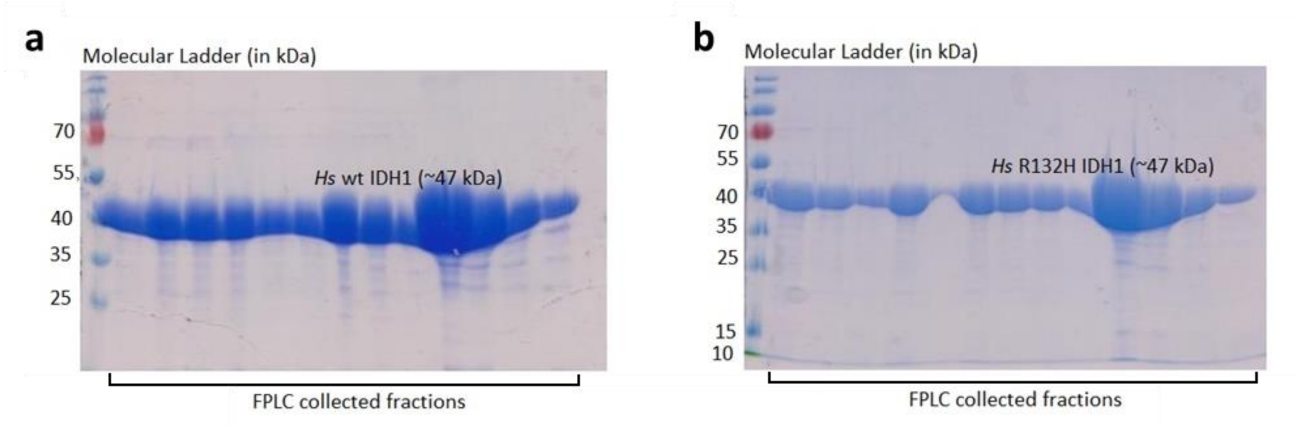

Supplementary Figure S19. **SDS-PAGE analyses of *Hs* wt IDH1 and R132H IDH1 as purified by FPLC.** Lane 1 – molecular markers (PageRuler Prestained Protein Ladder 10-170 kDa, ThermoFisher Scientific). Gels were stained with Coomassie blue.

Supplementary References

1. Cascón, A. *et al.* Whole-Exome Sequencing Identifies MDH2 as a New Familial Paraganglioma Gene. *J. Natl. Cancer Inst.* **107**, djv53 (2015).
2. Cardaci, S. & Ciriolo, M. R. TCA Cycle Defects and Cancer: When Metabolism Tunes Redox State. *Int. J. Cell Biol.* **2012**, 1–9 (2012).
3. Pasini, B. & Stratakis, C. A. SDH mutations in tumorigenesis and inherited endocrine tumours: lesson from the phaeochromocytoma-paraganglioma syndromes. *J. Intern. Med.* **266**, 19–42 (2009).
4. Mondesir, J., Willekens, C., Touat, M. & de Botton, S. IDH1 and IDH2 mutations as novel therapeutic targets: current perspectives. *J. Blood Med.* **7**, 171–80 (2016).
5. Maguire, M. E. & Cowan, J. A. Magnesium chemistry and biochemistry. *Biometals* **15**, 203–10 (2002).
6. Ma, R. & Yun, C. H. H. Crystal structures of pan-IDH inhibitor AG-881 in complex with mutant human IDH1 and IDH2. *Biochem. Biophys. Res. Commun.* **503**, 2912–2917 (2018).
7. Levell, J. R. *et al.* Optimization of 3-Pyrimidin-4-yl-oxazolidin-2-ones as Allosteric and Mutant Specific Inhibitors of IDH1. *ACS Med. Chem. Lett.* **8**, 151–156 (2017).
8. Deng, G. *et al.* Selective inhibition of mutant isocitrate dehydrogenase 1 (IDH1) via disruption of a metal binding network by an allosteric small molecule. *J. Biol. Chem.* **290**, 762–774 (2015).
9. Pusch, S. *et al.* Pan-mutant IDH1 inhibitor BAY 1436032 for effective treatment of IDH1 mutant astrocytoma *in vivo*. *Acta Neuropathol.* **133**, 629–644 (2017).
10. Okoye-Okafor, U. C. *et al.* New IDH1 mutant inhibitors for treatment of acute myeloid leukemia. *Nat. Chem. Biol.* **11**, 878–886 (2015).
11. Dang, L. *et al.* Cancer-associated IDH1 mutations produce 2-hydroxyglutarate. *Nature* **462**, 739–744 (2009).
