# Supplementary material for: Roles of metal ions in the selective inhibition of oncogenic variants of isocitrate dehydrogenase 1
Source: Commun Biol. 2021 Nov 1;4:1243. doi: 10.1038/s42003-021-02743-5 (PMC8560763; doi:10.1038/s42003-021-02743-5)
Supplement: Supplementary file 3 — Description of Additional Supplementary Files [file 42003_2021_2743_MOESM3_ESM.pdf]

Description of Additional Supplementary Files

File name: Supplementary Data 1

Description: Source data underlying the graphs in the main figures/table.
