# Supplementary material for: Roles of metal ions in the selective inhibition of oncogenic variants of isocitrate dehydrogenase 1
Source: Commun Biol. 2021 Nov 1;4:1243. doi: 10.1038/s42003-021-02743-5 (PMC8560763; doi:10.1038/s42003-021-02743-5)
Supplement: Supplementary file 5 — Reporting Summary [file 42003_2021_2743_MOESM5_ESM.pdf]

nature research

Corresponding author(s): CJ Schofield

Last updated by author(s): Sep 25, 2021

Reporting Summary

Nature Research wishes to improve the reproducibility of the work that we publish. This form provides structure for consistency and transparency in reporting. For further information on Nature Research policies, see our Editorial Policies and the Editorial Policy Checklist.

Statistics

For all statistical analyses, confirm that the following items are present in the figure legend, table legend, main text, or Methods section.

n/a	Confirmed	
☐	☒	The exact sample size (*n*) for each experimental group/condition, given as a discrete number and unit of measurement
☐	☒	A statement on whether measurements were taken from distinct samples or whether the same sample was measured repeatedly
☒	☐	The statistical test(s) used AND whether they are one- or two-sided<br>*Only common tests should be described solely by name; describe more complex techniques in the Methods section.*
☒	☐	A description of all covariates tested
☒	☐	A description of any assumptions or corrections, such as tests of normality and adjustment for multiple comparisons
☐	☒	A full description of the statistical parameters including central tendency (e.g. means) or other basic estimates (e.g. regression coefficient) AND variation (e.g. standard deviation) or associated estimates of uncertainty (e.g. confidence intervals)
☒	☐	For null hypothesis testing, the test statistic (e.g. *F*, *t*, *r*) with confidence intervals, effect sizes, degrees of freedom and *P* value noted<br>*Give P values as exact values whenever suitable.*
☒	☐	For Bayesian analysis, information on the choice of priors and Markov chain Monte Carlo settings
☒	☐	For hierarchical and complex designs, identification of the appropriate level for tests and full reporting of outcomes
☒	☐	Estimates of effect sizes (e.g. Cohen's *d*, Pearson's *r*), indicating how they were calculated

Our web collection on statistics for biologists contains articles on many of the points above.

Software and code

Policy information about availability of computer code

Data collection	No specialist code was used
Data analysis	Only commercially available programs were used, e.g. Microsoft Excel, Graphpad Prism.

For manuscripts utilizing custom algorithms or software that are central to the research but not yet described in published literature, software must be made available to editors and reviewers. We strongly encourage code deposition in a community repository (e.g. GitHub). See the Nature Research guidelines for submitting code & software for further information.

Data

Policy information about availability of data

All manuscripts must include a data availability statement. This statement should provide the following information, where applicable:

- Accession codes, unique identifiers, or web links for publicly available datasets
- A list of figures that have associated raw data
- A description of any restrictions on data availability

The authors declare that the data supporting the findings of this study are available within the paper and its supplementary figure files.

Field-specific reporting

Please select the one below that is the best fit for your research. If you are not sure, read the appropriate sections before making your selection.

☒ Life sciences ☐ Behavioural & social sciences ☐ Ecological, evolutionary & environmental sciences

For a reference copy of the document with all sections, see nature.com/documents/nr-reporting-summary-flat.pdf

Life sciences study design

All studies must disclose on these points even when the disclosure is negative.

Sample size	The number of biochemical and biological cell sample replicates in this study was determined by experience. 3 technical replicates were done in all biochemical and biophysical experiments in a plate format. NMR and non-denaturing MS experiments were done in a single replicate on one day; this was reproduced independently on separate days, and with two different inhibitors wherever applicable. Given the exploratory nature of untargeted metabolomics, where, by definition a large range of analytes are of interest across a wide concentration range that likely exceeds the dynamic range of the analytical system, it is not possible to calculate the number of replicates required. 3 tissue cultures replicates were used for each experimental group. This sample size was determined through trial and error.
Data exclusions	In general no data was excluded from the datasets discussed in this study unless explicitly described in the text.
Replication	Biochemical and biophysical experiments were successfully reproduced on separate days. As described in the manuscript the cell studies presented were replicated on a number of occasions over a 7 month period with similar outcomes.
Randomization	All sample analyses were randomized.
Blinding	Once data were generated by the mass spectrometer systems they were not blinded to the analyst. It was necessary for the data processing and statistical analysis to have knowledge of the experimental groups.

Reporting for specific materials, systems and methods

We require information from authors about some types of materials, experimental systems and methods used in many studies. Here, indicate whether each material, system or method listed is relevant to your study. If you are not sure if a list item applies to your research, read the appropriate section before selecting a response.

Materials & experimental systems

n/a	Involved in the study	
☒	☐	Antibodies
☐	☒	Eukaryotic cell lines
☒	☐	Palaeontology and archaeology
☒	☐	Animals and other organisms
☒	☐	Human research participants
☒	☐	Clinical data
☒	☐	Dual use research of concern

Methods

n/a	Involved in the study	
☒	☐	ChIP-seq
☒	☐	Flow cytometry
☒	☐	MRI-based neuroimaging

Eukaryotic cell lines

Policy information about cell lines

Cell line source(s)	The LN18 cell line was purchased from ATCC
Authentication	Authentication from ATCC was replied upon
Mycoplasma contamination	The cell line was regularly tested for mycoplasma contamination; all tests were negative.
Commonly misidentified lines (See ICLAC register)	None we are aware of.
